# Is innovative workforce planning software the solution to NHS staffing and cost crisis? An exploration of the locum industry

**DOI:** 10.1186/s12913-018-2989-x

**Published:** 2018-03-20

**Authors:** Iakovos Theodoulou, Akshaya Mohan Reddy, Jeremy Wong

**Affiliations:** 1 King’s College London, Guy’s Hospital, Great Maze Pond, London, SE1 9RT UK; 2 University of Leicester, Centre for Medicine, 15 Lancaster Road, Leicester, LE1 7HA UK

**Keywords:** Healthcare, Workforce planning, Recruitment, Mobile technology, Innovation

## Abstract

**Background:**

Workforce planning in the British healthcare system (NHS) is associated with significant costs of agency staff employment. The introduction of a novel software (ABG) as a ‘people to people economy’ (P2PE) platform for temporary staff recruitment offers a potential solution to this problem. Consequently, the focus of this study was twofold – primarily to explore the locum doctor landscape, and secondarily to evaluate the implementation of P2PE in the healthcare industry.

**Methods:**

Documentary analysis was conducted alongside thirteen semi structured interviews across five informant groups: two industry experts, two healthcare consultants, an executive director, two speciality managers and six doctors.

**Results:**

We found that locum doctors are indispensable to covering workforce shortages, yet existing planning and recruitment practices were found to be inefficient, inconsistent and lacking transparency. Contrarily, mobile-first solutions such as ABG seem to secure higher convenience, better transparency, cost and time efficiency. We also identified factors facilitating the successful diffusion of ABG; these were in line with classically cited characteristics of innovation such as trialability, observability, and scope for local reinvention. Drawing upon the concept of value-based healthcare coupled with the analysis of our findings led to the development of Information Exchange System (IES) model, a comprehensive framework allowing a thorough comparison of recruitment practices in healthcare.

**Conclusion:**

IES was used to evaluate ABG and its diffusion against other recruitment methods and ABG was found to outperform its alternatives, thus suggesting its potential to solve the staffing and cost crisis at the chosen hospital.

## Background

Healthcare systems such as the English National Health Service (NHS) clearly recognise the potential of smartphone apps to improve healthcare access and patient health [1]. While most of the 165,000 available health apps are aimed at patients [2], many are increasingly designed to assist healthcare professionals in work-related tasks.

A smartphone app, known henceforth as ABG for confidentiality, is one such example. ABG acts as an interface facilitating direct communication between doctors looking to do locum shifts and hospitals with vacant shifts. Speciality managers, who organise staff rotas within their department can advertise available shifts and its details on an online, mutually accessible notice board from which doctors with the app can view and apply to cover shifts. The premise of this mobile platform is to help healthcare providers decrease their spending on recruitment agencies and streamline workforce planning by uncoupling agencies from the recruitment process.

In October 2016, ABG was adopted by Kappa Hospital (KH) as a novel, mobile-first technology solution to reduce employment of agency locum doctors and maximise utilisation of the Trust’s ‘internal staff bank’. KH is a pseudonym for the Trust adopted for confidentiality purposes. The concept of ABG is based on the ‘people to people economy’ (P2PE) model, where self-employed individuals offer services through internet-based matching platforms [3]. Disruptive innovations facilitating P2PE reduce transaction costs and mitigate information asymmetry [4]. As described by Hwang and Christensen [5], these products are “simpler, more convenient and more affordable” and are usually introduced by new market entrants who eventually displace incumbent firms. A most recent manifestation of this phenomenon includes the taxi industry and Uber [6].

Rogers’s [7] definition of innovation as “an idea...that is perceived as new by unit[s] of adoption”, implies that for ABG’s success will depend on its diffusion [7]. Classical diffusion theory states that innovations follow an ‘S curve’, the beginning of which is characterised by a lag phase, where early adopters subsequently influence the ‘early majority’ until a critical mass is reached [7]. Rogers’s linear model is challenged by Greenhalgh et al. [8] who posit that diffusion is “complex, iterative, organic and untidy”, with the value of the innovation changing over time in accordance with the context and its interpretation by people.

Research in healthcare innovation support the latter notion as the characteristics of innovations constantly interact with contextual factors, even after the adoption stage [9, 10]. For example, research in electronic health records suggests that lack of support and follow-up training is a usual cause of ‘un-adoption’ [11, 12]. Variable adoption rates between local providers which have been attributed in part to a lack of leadership in sustaining innovations beyond their pilot phase, has meant that despite the abundance and potential of new technologies to improve healthcare delivery, the NHS has failed to fully capitalise on these [13, 14]. Nevertheless, we note that research into NHS has mainly been restricted to singular facets for analysis [15, 16] and thus, falls short of exploring the mechanisms of local adoption behaviour [17]. Therefore, conducting an in-depth analysis of ABG’s diffusion in KH may facilitate attempts to accelerate its adoption and diffusion on a larger scale.

The innovation is in line with Porter and Teisberg’s ‘Value Based Healthcare’ (VBH) [18] which proposes that the incorporation of interoperable IT systems that provide seamless communication among involved parties leads to increased value in healthcare. Since locum recruitment represents an integral element of the human resource management (HRM) component of the value chain, it may be argued that the adoption of ABG is a significant step towards VBH. While previous research has primarily focussed on the effect of mobile platforms on the ‘primary’ activities of the VBH model [19, 20] this study is the first to explore their effects on its ‘support’ activities. ABG’s pilot scheme at KH was the focus of this study.

Although agencies do respond to hospitals’ requirements for clinical staff, from 2009 to 2015, NHS expenditure on agency staff had increased from £2.2 billion to £3.3 billion [21]. More recently, healthcare providers (Trusts), have come under pressure to reduce their spending on recruitment agencies. The regulatory body NHS Improvement (NHSi) highlighted that agency spending is deteriorating Trusts’ finances [22], contradicting the traditional rationale that agencies reduce costs and time spent securing and enforcing contracts [23, 24]. Undersupply of workers seems to be an important driver of agency staff use in the NHS [25, 26]. The need to achieve efficiency savings coupled with the Francis Report calling for safe staffing levels have forced Trusts to understate staffing needs, paradoxically leading to staffing gaps and unsustainable increase in demand for agency workers [27]. Inspired by these challenges, this study aimed to examine the implementation of ABG and compare it with concurrent locum doctor recruitment strategies.

Whilst newly introduced regulatory measures on agency spending have enabled NHS achieve £1 billion worth of savings, many Trusts are still struggling to meet these requirements [28, 29] with 85% of Trusts having exceeded agency hourly caps within 5 months of their introduction [30]. Limited progress was also noted in relation to the secondary objective of the cap regulations, which was to dissuade healthcare professionals from joining agencies in preference to NHS staff banks [31]. While studies do suggest that part-time agency work is mainly precipitated by non-wage factors such as flexibility and work pressures in NHS [32–34], their focus has been on pharmacists and nurses with the perspectives of locum doctors remaining unexplored [34]. Given the ambiguity of their impact in healthcare [26, 35] this study aimed to explore doctors’ views of temporary locum work compared to full-time employment; secondly, to examine the formal and informal locum doctor recruitment strategies adopted by speciality managers in the Trust; and lastly, to evaluate the implementation of ABG as a recruitment strategy, focusing on themes of efficiency, functionality and quality assurance.

## Methods

### Study design

Gaining insight into a subject for which there is little empirical evidence made a qualitative approach to our study natural. This was reinforced by the fact that ABG had not been piloted long enough at the time of data collection to yield significant data for quantitative analysis. To conduct an in-depth user evaluation of ABG, the study used thirteen semi-structured interviews (SSI) with its main stakeholders. Documentary analysis of publicly available board meetings and Care Quality Commission (CQC) reports were also used to gain additional contextual information.

### Study settings and sampling

KH was rated as ‘good’ by the CQC in 2013 across all five inspection areas, such as leadership and responsiveness. Staff shortages and overcapacity were identified as shortfalls for the Trust, particularly in medical wards where they impacted upon safety protocols.

We engaged in non-probability sampling to increase perspective heterogeneity of ABG’s pilot. First, purposive sampling was used to identify two industry experts closely tied to ABG (I1, I2) and two independent, UK based strategy consultants in digital healthcare (HC1, HC2). They were selected for their specialist knowledge of locum work, recruitment strategies and healthcare innovation.

This was followed by three rounds of snowball sampling which provided us with an Executive Director of the Trust (ED1); two speciality managers using ABG (SM1, SM2); and six doctors using the app (JD1–6). Informants were recruited on the basis of having had experience of different recruitment strategies and the wider locum industry. Recruitment was ceased upon reaching a point of data saturation.

### Data collection

Data collection occurred in the months of February and March 2017 when the pilot had been established for almost six months. Ethical approval was secured in January 2017 from King’s College London Ethics Committee. All informants gave both verbal and written informed consents to participate in this study prior to interviews. Nine of the interviews were face-to-face and four were by telephone due to scheduling issues. The interview sites varied according to convenience for the interviewees, though quiet and private settings were selected to ensure minimal distraction and maximal anonymity [36]. Interviews lasted an average of 45–60 min. With informed consent, twelve of the thirteen interviews were recorded and transcribed verbatim within 12 h of the interview to ensure reliable data analysis [37]. One interview was not recorded as the informant denied consent to record. During this interview, a second interviewer was responsible for taking detailed notes to ensure the interviewee’s responses could still contribute to data analysis. The transcriptions were then cross-checked against the voice recording by other researchers to ensure accuracy.

A distinct interview schedule tailored to each informant group formed an aide-memoire for the researchers, which were developed from initial informal conversations with key stakeholders and a preliminary literature review. Multiple researchers participated in the interviewing process which helped ensure analyst triangulation and minimise potential bias [38]. Two researchers were present per interview: one led the interview whilst the other ensured adequate coverage of the interview schedule and followed up on important trajectories as they emerged. Member checking techniques such as paraphrasing and summarising were utilised during the interview to minimise ‘distortion’ [39].

### Data analysis

NVIVO 11 was used to assist in thematic analysis of the interview transcripts. We found a mixed deductive and inductive approach [40] useful in generating 20 germinal parent nodes by allowing our objectives to guide the generation of codes whilst remaining open to new themes generated from the data. All transcripts and documents were coded by all researchers independently to increase analyst triangulation [38]. A constructivist approach [41] enabled us to accommodate complementary and contradictory data by accepting diverse socially constructed realities of locum work and evolving recruitment processes. Analyst triangulation and constructivism helped guard against selectivity in the use of data [42].

Each round of analysis resulted in coding adjustments as we noticed shifts in patterns over time. These changes were discussed to minimize ambiguity of codes and augment the reliability and rigour of our analysis [38, 43]. Resulting themes were organized into three overarching vignettes: ‘locum work’, ‘workforce planning’ and ‘workforce planning technology’. These follow the framework laid out by our objectives and form the skeleton for the results section.

## Results

### Locum work

Many of the informants agreed that locum work is an under-explored topic that is often depicted inaccurately in the media. Career development, job nature and patient care were angles explored when asked about what locum work entails.

#### Job description

As indicated by both the literature review and the documentary analysis, no single description can encapsulate the multi-faceted nature of locum work. Informants have portrayed locum work highly variably in terms of pay, contract length and impact on patient care. Doctors either cover shifts impromptu, or enrol on longer-term contracts with the latter being perceived as ‘safer and preferable’ for patient care, giving doctors time to adapt to unfamiliar environments.

*JD5: “Long term locums are there for months…they get very good at their job as they become part of the team”*

This form of ‘locuming’ is usually more common with doctors at their ‘F3’ year – a year spent outside traditional training pathways. JD2 argues that ‘F3’ allows doctors to consider their choice of speciality more carefully, while enriching their portfolios. Despite the attractiveness of this career option, a different doctor expresses his scepticism about ‘F3’, describing it as a state of uncertainty.

*JD2: “at some point you don’t have a shift to do, you are waiting for the next one, and you are in transit… the satisfaction struggles to be the same.”*

We found job satisfaction amongst locums to be highly variable. Some admit that working solely as a locum cannot be uncoupled from a lack of career progression, ultimately leading to ‘motivational burnout’. Despite the attractive pay patterns, some doctors generally consider ‘locuming’ an unsustainable career option often defeated by an overarching desire to attain a secure position.

*JD4: “I would prefer to be in a training programme definitely. Working as a locum doesn’t let you progress career wise – I like to be learning new things.”*

Some shorter-term locums attribute their dissatisfaction to their inability to follow up patients. For example, while successful patient recovery is a frequent source of job satisfaction, short-term locums rarely get the chance to experience this. Moreover, such locums rely on locum agencies for finding jobs, the recruitment process of which is frequently a source of frustration. According to JD1, all these issues are rarely associated with longer-term locum posts.

*JD2: “You don’t see that you’ve diagnosed correctly or that the patient received the right treatment and they got better and went home…so you miss out on that as a locum.”*

**CONCLUSION 1.1**: Locum job satisfaction is highly variable and dependent on contextual factors including the duration of the contract.

#### Motivational factors

We identified many factors determining whether one will undertake locum work. For example, locum hourly rates supersede those of full-time contracts, and pay-centric doctors appear happy to avail of such enticing pay rates. However, there is also solidarity across all doctors’ interviews in that the decision to do locum work is not solely monetarily driven. Indeed, locum work seems to be the product of multiple non-wage determinants such as convenience, logistics and compatibility with posts.

*JD5: “…it’s much more lucrative than working in an NHS-contracted job by two or three times.”*

*JD3: “…there are more elements other than how much you get paid – how comfortable you are working in certain posts…how easy it is to book the shift…how much you trust the person who gives you the job...”*

Stemming from the fact that the early training programmes limit doctors’ exposure to certain specialities, supplementary ‘locuming’ provides them the opportunity to attain additional skills they have not had the chance to do so previously. Some informants admit that without such opportunities, their choice of speciality would be less informed. This contrasts earlier findings where locuming was found to hinder career progression. Whilst working in new environments and learning new skills is attractive, these benefits mostly come with longer-term locuming.

*JD3: “Working as a locum provides you with the flexibility to work in specialties that you may not have had experience in.”*

**CONCLUSION 1.2**: Locum work is viewed positively from a doctor’s perspective because of the additional income and the career opportunities it offers.

#### Challenges of locum work

Most of the system inefficiencies doctors face relate to short-term locum posts where they are asked to cover shifts with very short notices. Working at a new hospital for the first time can be alienating as doctors need to quickly adapt and work in unfamiliar environments in the absence of ‘camaraderie’ benefits. Uncertainty encompasses many aspects, from knowing where to go and who to report to, to more fundamental issues such as accessing buildings. Nevertheless, some comments help clarify that these challenges can be overcome with time, for example by working in the same hospital and gaining the much-needed acquaintance. Collectively, these findings suggest insufficient hospital induction practices are the most important deterrent to undertaking locum work.

*JD2: “If information was made more available then it would improve the process.”*

Practical inefficiencies often prevent a smooth completion of shifts due to unnecessary delays and logistical ‘headaches’. Some may affect patient care as they prevent doctors from ordering examinations, tracking a patient’s progress or communicating with other team members. Lastly, an internal resentment among native staff about the high pay rates of external locums, finds expression in JD4’s statement below.

*JD5: “Sometimes you don’t even get a login or a card. Then you can’t order any blood test or look at results…”*

*JD4: “when someone comes in, with little experience of the speciality and gets paid a much higher rate than someone on a training programme… that can make people angry”*

**CONCLUSION 1.3**: Locum practices are currently highly variable and inefficient.

### Workforce planning

We found that locum doctors are indispensable for covering vacancies; without whom according to JD3 ‘the NHS could collapse’. Consequently, we collected views about the planning strategies used by speciality managers before the adoption of ABG. This allowed us to reveal key sources of inefficiencies and to invite our informants to suggest solutions. An important dimension of the recruitment process is that of information exchange. Table 1 summarizes the information required by doctors and managers respectively to make informed decisions, which, as we conclude, is seldom exchanged effectively.

**Table 1:** Information requirements revealed by the interviews

For locum doctors:	For speciality managers:
Speciality required	Speciality of applying doctor
Grade requirements	Grade of applying doctor
Duration of post	Availability of doctors
Who to report to	Doctor contact details
Access to hospital buildings/wards	Photo of person booked for shift
Whereabouts/map of hospital	Pre-employment checks
Access to patient records	Previous quality issues
Hourly rates and payment methods	Knowledge of the real market rate

#### Inefficiencies

On asking our informants to evaluate previous strategies, a sense of frustration is expressed by both doctors and managers. Indeed, recruitment practices are described as ‘outdated’, ‘reactive’, ‘time-consuming’ and ‘inefficient’. This theme’s findings are organised under the main workforce planning tools: ‘internal staff banks’ and ‘external locum agencies’.

##### Internal staff banks

Among the various strategies adopted by speciality managers, the internal staff bank (also referred to as ‘doc bank’) has been their most preferable method, albeit rather inefficient and time-consuming. This ‘in-house agency’ comprises of internal doctors who have previously covered vacancies and is the closest to a planning strategy that managers used to plan rotas.

*SM2: “We had a spreadsheet with a list of doctors. If we needed one, the first thing we did was to send an email to all of our ‘doc bank’ staff...”*

Reinforcing the preference for the ‘doc bank’ was a top-down initiative from the Trust’s board to increase the use of internal bank staff and decrease the use of agency staff. As the Trust’s management elaborates, the motive was partly financial and partly safety-related. They explain that doctors already enlisted in the Trust have gone through all essential pre-employment checks and thus constitute safer choices.

*SM2: “We would like to use our ‘doc bank’ because we know the doctors, they’ve been checked, we know they’re not going to be a risk to us. With agencies you can only get a vibe from what the CV says.”*

Another benefit of utilising internal staff banks is that in doing so the Trust contributes to staff development by giving doctors more training opportunities and experiences.

*ED1: “We are investing in our own bank doctors to develop them, to become one day consultants or leaders. It’s an investment for everyone.”*

Previous planning strategies are articulated more accurately by I1 who worked at KH in the past. He describes it as merely an upgrade of handwritten records to a complicated spreadsheet. Despite the managers’ strong preference for the ‘doc-bank’, many disadvantages can be identified. It is time-consuming, prone to errors, and most importantly, it often fails to cover the staffing gaps. As explained by ED1, speciality managers are often short of options since the ‘doc-bank’ list is outdated which prevents them from accessing new Trust doctors. From a doctor’s perspective, deciding to do locum work is a similar story: from booking a shift to completing it and receiving payment, it is a long, frustrating and unreliable process. Examples include extremely short notice; a general lack of transparency and poor pay management.

*JD1: “You would get a panicky email when the shift was due to start…or a speciality manager running around the ward trying to find doctors.”*

Furthermore, one of our informants provides ample evidence to support the doctor-manager chasm that arises because of recruitment inadequacies. On tracing the underlying causes of the staff shortages further, we analysed existing board minute reports and deduced a relatively high staff turnover rate in the Trust; an issue that has been a recurrent subject of discussion in board meetings, particularly in 2016.

*JD5: “The manager goes home at 5pm having not filled the shift and she doesn’t really care because it’s not part of her job...but because they’re not doctors, they don’t realise that the hospital works 24/7 and that the rest of us just take on the slack.”*

##### External agencies

When the ‘doc bank’ proves insufficient, speciality managers turn to agencies. They will then be faced with a two-fold pressure: one to keep within their pay budget, and two, to recruit doctors of the appropriate grade and speciality. Managers discuss that rarely are they able to achieve both these objectives within the short notice that is given to them. All this makes working with agencies extremely challenging and inefficient.

*SM1: “you call agencies, you call people who call agencies – so there’s middlemen for middlemen, and then you just wait for someone to get back.”*

*SM2: “If we didn’t get responses [from the internal bank staff] we would then ring around the agencies”*

A manager argues that the lack of access to information relating to agency staff makes their job uncertain. Although they appear cognizant of the ability of agencies to financially exploit them, they admit this is inevitable since they have no control over the locum market. Therefore, the Trust’s financial interests are often compromised at the expense of a more important objective – to meet staffing requirements.

*HC1: “agencies are saying ‘Well, we can’t find anyone, unless you put the price up a little bit’ and the hospital has no idea how many people are on the market, so they agree.”*

Doctors’ experiences of locum agencies are highly varied, with some describing them as ‘irritating’ and ‘not the ideal’ job-searching tool. In contrast, more pay-centric doctors who see locum work as an extra source of income seem to prefer agencies for accessing jobs. Others simply like the fact that agencies take all the administrative burden of finding jobs.

*JD2: “The locum agencies themselves will harass you. You will generally get two to three calls day. It is annoying…you end up blocking them!”*

**CONCLUSION 2.1:** Information exchange is a key element of locum work, yet it often fails to happen optimally through current recruitment practices.

### Workforce planning technology

To produce comprehensive contextual analysis, we explored our informants’ views of ABG and its potential to solve existing inefficiencies. Despite still being at its early stages, we found strong support about its potential to solve what appears to be a simple yet resource-draining problem. A documentary analysis of a most recent CQC report, revealed two main challenges faced by the HRM both of which seem to have contributed to the receptivity to ABG. Staffing shortages and the associated undermining of quality of care were the most important of these.

### User evaluation

**Fig. 1:**
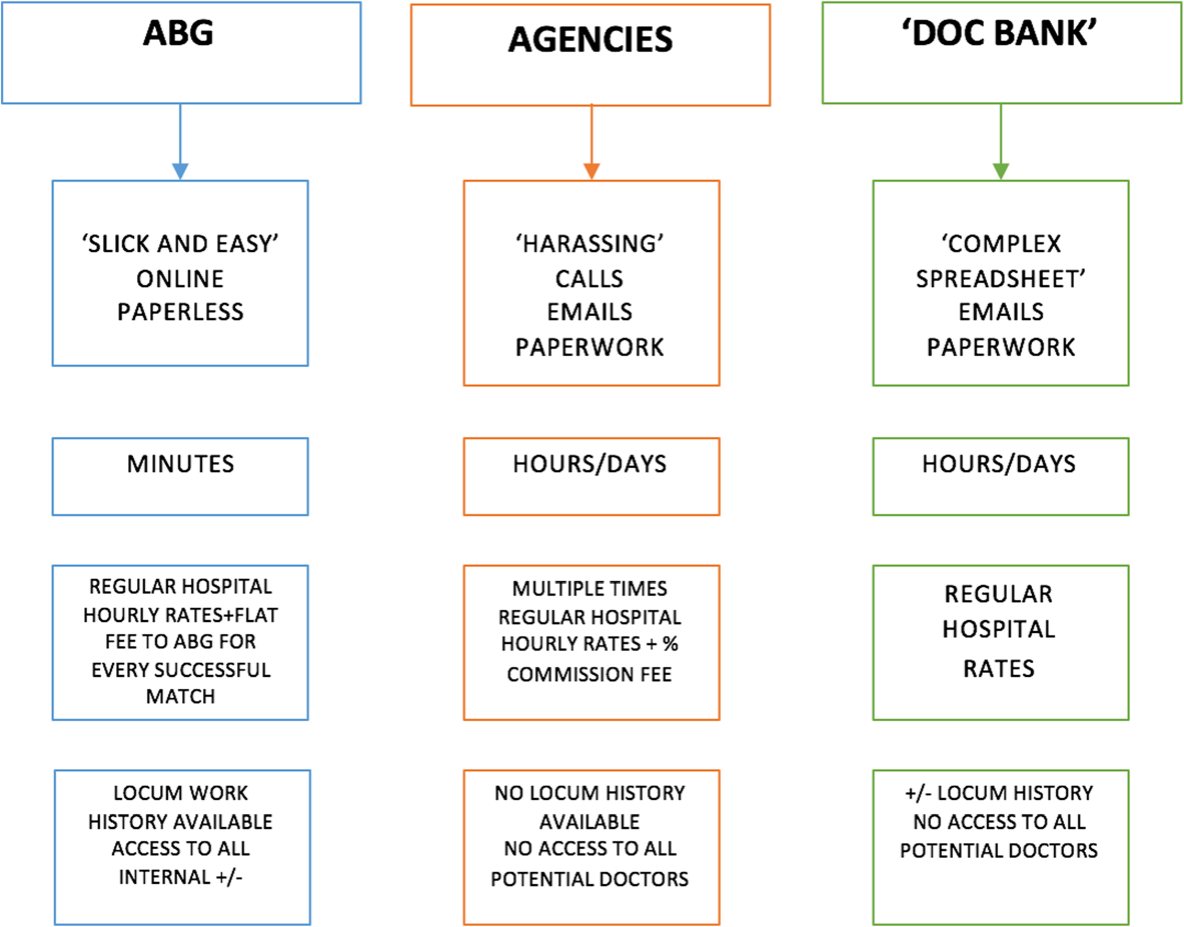
Gathered descriptions of the three recruitment methods

On asking doctors from the hospital to share their experiences with ABG, the findings were overwhelmingly positive. We provide a summary of our findings regarding the description of the three recruitment strategies including ABG, in Fig. 1.

#### Ease of use

As illustrated through earlier themes, previous recruitment strategies are highly inefficient and oftentimes complex. On the contrary, ABG is designed in a way that makes things more streamlined. Doctors feel more in control of their own work rather than relying on the often-bothersome agencies, or highly bureaucratic hospital administration. The ability to book shifts themselves, allows them to choose when to engage with the app. Similarly, planning for managers has become more flexible as the software can be managed from virtually anywhere. In exploring the contextual factors that made the adopters more receptive to this solution, many discuss the congruity of the smartphone concept with wider lifestyle trends.

*SM2: “Everything you do these days is online…you can do the same with booking shifts.”*

*HC1: “What really impressed me about this application is that it’s very simple to use, it’s very user-friendly”*

#### Administrative efficiency

The findings support the long-held view that mobile-first technology can play a bigger role in healthcare systems. The informants discuss the efficiency that the new platform provides, both in terms of time and in terms of overcoming the logistical challenges that were noted earlier. The application lessens managers’ everyday workload, thus allowing them to dedicate more time to long-term planning. Making paperwork redundant is another advantage as it solves previously noted inefficiencies.

*JD5: “The app tracks how and what you’re paid with all your timesheets in, that’s just such a huge benefit…for managers as well…they don’t have to track loads of bits of paper.”*

##### Trust objectives

Most importantly, informants discuss the catalytic role of the new software in helping the Trust meet its objectives. Although budgets do influence hospital recruitment preferences, they are of secondary importance to patient safety. In relation to the latter, ABG allows speciality managers to identify doctors of the desired grade and speciality, engage in proactive planning and ensure care quality. ABG also allows managers to flag any doctors whom they have concerns about and investigate their performance further. Direct communication of doctors with the Trust means that both doctors and managers make informed decisions due to improved information exchange.

*I2: “The hospital is using its own doctors more. They had a 300% increase in terms of their own doctors filling the vacancies.”*

*ED1: “the first 4 months we saved around £21 000 as a Trust.”*

Locum recruitment seems to have been an opaque process with the main stakeholders often deprived of important information. The improvements in transparency are discussed by HC1; he argues ABG makes recruitment “*more transparent, easier and convenient*” and discusses the possibility of agencies becoming obsolete. Managers and directors, acting as the main commissioners of agencies, describe previous processes as highly blurred, obstructing informed decision-making and accountability. Contrarily, they view ABG as a promising pathway to restoring the much-needed transparency

*HC2: “if hospitals shift to this app, there’s a stark possibility that agencies aren’t going to be needed.”*

*HC1: “pay-centric locum doctor have been using agencies to get higher rates - I think ABG will damage that”*

##### Better ‘locuming’

As opposed to the challenges discussed earlier, doctors describe locuming through ABG as a smoother experience. This is primarily due to more information being available to them prior to actual shifts, which increases their confidence in performing their role. However, although more information is indeed available, it is not always easily accessible. For example, JD4 admitted he has not yet been able to access the information, thus indicating the need to educate users. Our findings also suggest that the application’s filtering properties personalise locuming and increase safety by optimising job searches using criteria that best suit the preferences and competencies of doctors - for example, by speciality or grade. This novel feature also contributes to career development as it enables exposure to desirable specialties.

*JD2: “managers can give you information through the app about where you’re going, what kind of work you’ll be doing, who to report to…So any problems, you know who to approach.”*

### Diffusion of innovation

To evaluate ABG’s diffusion and thus uncover its main promoters and barriers, we explored our informants’ thoughts on the efficiency with which it is spreading across and within hospitals.

#### Word of mouth and observability

As supported by HC1, awareness about the existence of the innovation is a key component for its success. Having established the importance of communication channels, the most frequently cited diffusion pathway was through ‘word of mouth’. More centralised, Trust-wide initiatives are also pursued by the Trust to raise awareness and encourage the use of the app.

*ED1: “If the product is good and it is saving them money, I think it is something that they [HR directors] will ultimately talk about with their colleagues in another hospital.”*

Another factor instilling confidence in ABG is the tracking of performance indicators, which are used to deduce cost savings and compare internal staff utilisation under different strategies. Whilst robust service evaluation usually requires longer trial runs, the early efficiency savings reassured managers of ABG’s capabilities.

*ED1: “We made them [managers] see the benefits of it - no time sheets…no pieces of paper going misplaced or missing.”*

#### Trialability

The opportunity to enter a pilot scheme without any contractual commitments, has been one of the key enhancers of its adoption and subsequent diffusion. We find that trialability is a promoter of adoption at both an organisational and individual level. Indeed, managers utilised the probation period to assess the technology, compare it with existing strategies and suggest modifications tailored to their needs.

*HC2: “Technically when a Trust buys software they make a huge upfront investment by paying annual support and maintenance. This is ‘pay as you use’ with no upfront investment”*

#### Barriers to diffusion

Failure to appreciate the indispensability of locum doctors is tantamount to ignoring the potential of this innovation to disrupt the locum industry and revolutionise workforce planning. Despite his optimism about ABG, HC2 expresses his reservations about its wider spread given the lack of formal dissemination policies in the NHS. Owing to the apparent inconsistencies, he supports that top down strategies could be beneficial. Another concern expressed by JD2, is the oftentimes problematic and fruitless translation of innovations from ‘bench to bedside’.

*HC2: “There is a lack of consistency…there’s nobody in NHS who’s going to say ‘Every Trust must use this technology’ - it doesn’t work like that. But, the outcome would be far better if that happened”*

**CONCLUSION 3:** Successful adoption and diffusion depends on word of mouth, the option of trialability, observability of outcomes and the scope for ‘local reinvention’.

### Information exchange system (IES) model

**Fig. 2:**
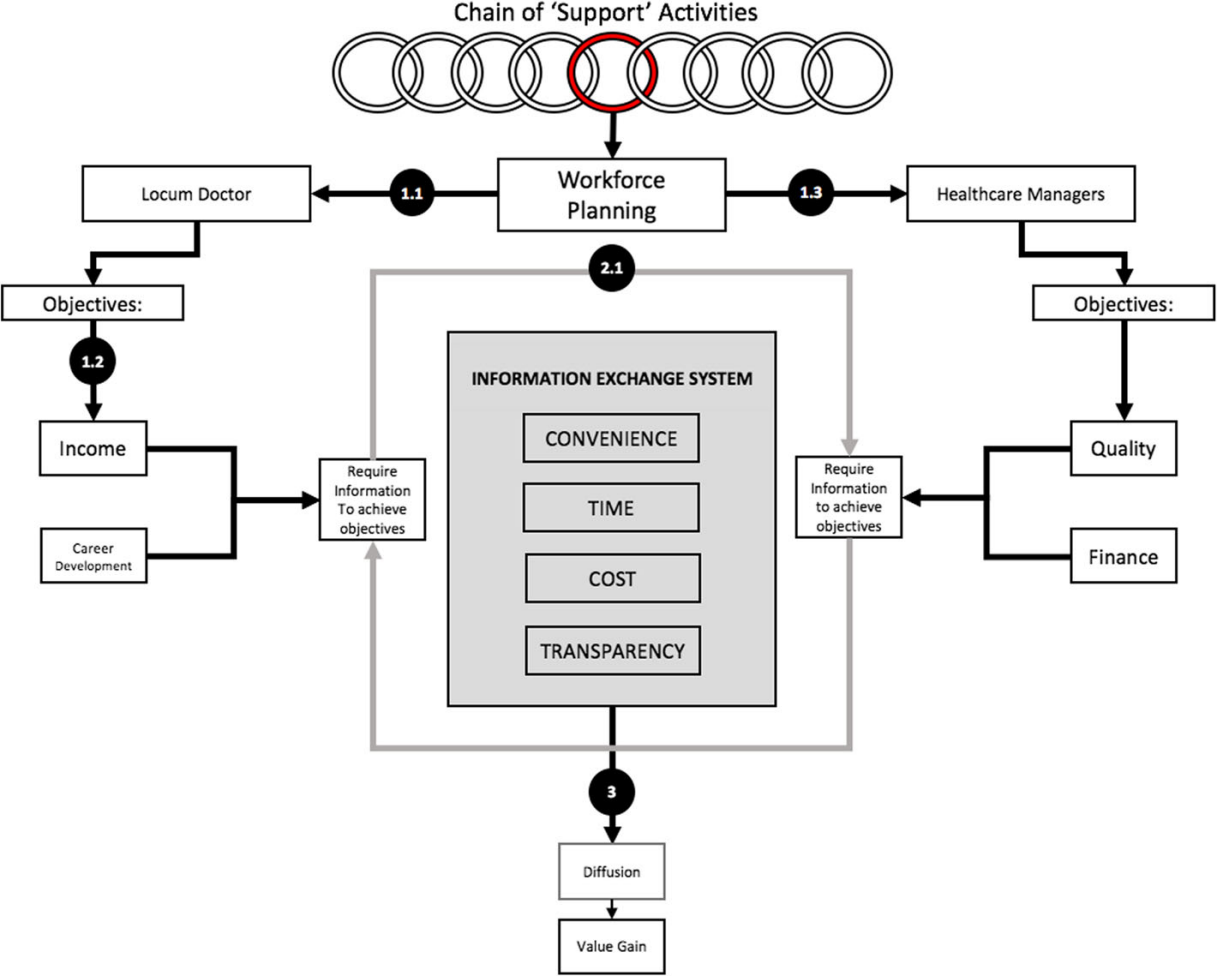
The IES model. Numbers in circles correspond to the conclusions made at the end of corresponding themes. Themes 1.1 and 1.2 shaped the role of locum doctors and 1.3 shaped the managers’ side. Theme 2.1 helped identify the parameters of the information exchange system and Theme 3 identified the importance of effective diffusion of practices that optimise the IES

After analysing the emergent themes, we integrated them in the IES model, a socially constructed system of information flow underpinning workforce planning in hospitals (Fig. 2). The IES encapsulates two main stakeholders, doctors and managers, whose interdependence and communication is pivotal for effective exchange of different information parameters (Table 2). Locum doctors are driven by income and career opportunities whilst managers focus on high-quality, financially sound solutions. For them to achieve their respective objectives a successful ‘negotiation’ through an information cycle must happen. As inferred from our thematic analysis, the efficiency of this cycle relies on four dimensions: convenience, time requirements, cost and transparency. These were identified by eliciting our informants’ views about factors inhibiting the current system.

**Table 2:** Assessment of the recruitment strategies based on the four IES dimensions

IES Dimensions	Traditional Staff Banks	External Locum Agencies	ABG
Convenience (1 = low, 2 = high)	1	1	2
Time Requirements (1 = high, 2 = low)	1	1	2
Transparency (1 = low, 2 = high)	2	1	2
Within budget (1 = no, 2 = yes)	2	1	2

Furthermore, the IES evaluation grid (Tables 3, 4 and 5), enables Trusts to evaluate alternative recruitment strategies and therefore make informed decisions regarding the adoption of new technologies. Employing a binary scale (1/2) and using the findings of our assessment (Fig. 1), we provide ratings for the three recruitment strategies employed to-date (Table 2). Acknowledging the fact that different departments may have different priorities with regards to the four dimensions, we have incorporated a weighing feature that enables evaluation tailored to their local needs.

**Table 3:** IES evaluation grid for Locum Agencies

	Recruitment method: Locum Agencies																
IES dimensions	Convenience				Time Requirement				Transparency				Cost within budget				
Score(delete as appropriate)	Low		High		Low		High		Low		High		No		Yes		
	1		2		2		1		1		2		1		2		
Relative importance for Trust/Department 1 = low, 4 = high	1	2	3	4	1	2	3	4	1	2	3	4	1	2	3	4	Total score
Score x Weight	2				1				4				3				10

**Table 4:** IES evaluation grid for Traditional Staff Banks

	Recruitment method: Traditional Staff Banks																
IES dimensions	Convenience				Time Requirement				Transparency				Cost within budget				
Score (delete as appropriate)	Low		High		Low		High		Low		High		No		Yes		
	1						1				2				2		
Relative importance for Trust/Department 1 = low, 4 = high	1	2	3	4	1	2	3	4	1	2	3	4	1	2	3	4	Total score
Score x Weight	2				1				8				6				17

**Table 5:** IES evaluation grid for ABG

	Recruitment method: ABG																
IES dimensions	Convenience				Time Requirement				Transparency				Cost within budget				
Score (delete as appropriate)	Low		High		Low		High		Low		High		No		Yes		
			2		2						2				2		
Relative importance for Trust/Department 1 = low, 4 = high	1	2	3	4	1	2	3	4	1	2	3	4	1	2	3	4	Total score
Score x Weight	4				2				8				6				20

## Discussion

To our knowledge, our study is the first to conduct a multi-level analysis of locum doctor employment. We assessed how innovative solutions can address a failing locum industry by evaluating the readiness of KH to adopt, disseminate and benefit from ABG. Inspired by Edmondson [44] who hypothesised healthcare failures often arise from ‘breakdowns in communication’, we used our primary data and Porter’s VBH [18] to develop the IES model described above. ABG (Table 5) is presented as a ‘technological fix’ within a ‘complex socio-technical system’ [45], as it successfully overcomes shortcomings of previous strategies (Tables 3 and 4) such as expense and opacity. Therefore, healthcare value attributed to workforce planning improvements does seem to depend on the efficiency of IES.

We stipulate that the perceived disequilibrium in terms of wage and levels of permanent employment are symptoms of a bilateral monopoly between the near-monopsony NHS and a monopolistic supply of doctors [46]. Likewise, the perceived need for additional career opportunities helps substantiate the widely-held belief that non-wage determinants have greater impact upon supply as opposed to the economic maximisation rationale. We conjecture that undertaking locum work may be a manifestation of an imperfect healthcare labour market that fails to maximise the utility of its constituents.

Whilst existing literature remains inconclusive regarding the effects of high locum dependency on patient safety, according to our informants, incidents of locum malpractice at KH mainly result from organisational inefficiencies as opposed to incompetence; this essentially suggests problematic IES. Ample evidence suggests that under existing recruitment strategies, doctors are deprived of information imperative for the safe performance of locum work in two ways. Firstly, the absence of information prior to signing up for a locum shift can lead to employment of doctors who are ‘unfit for purpose’. Secondly, highly variable induction practices often render locum doctors reliant on native staff for matters as simple as accessing patient records and admission to restricted areas. On busy wards this can lead to inefficiencies and compromised patient safety. We conclude that this IES opacity results in latent systemic conditions that lay the groundwork for medical errors [47]. We hereby stress the importance of prioritising locum induction before shifts and ensuring access to electronic and physical spaces. The transparent IES ABG provides enables the main stakeholders to make informed decisions leading to value improvements.

Agencies are often perceived as intermediaries with access to many more doctors, theoretically increasing the likelihood of finding a match fit for purpose. However, agency use also indicates inability to utilise internal human resources. Illegitimate manipulation of information flow by agencies renders the IES asymmetric, thus causing an overly chaotic locum market. Though traditionally intermediaries (agencies) claim to reduce the costs of acquiring information for both demanders (hospitals) and suppliers (doctors), our findings suggest that an over-reliance on agencies may have transformed them from cost shavers to cost-causers. The 3-month cost savings achieved by a single ward under ABG seems to be the first piece of evidence to reinforce the latter argument. Therefore, we conclude that short-term solutions offered by agencies are frequently to the detriment of longer-term financial objectives.

Porter’s VBH model emphasises the importance of restructuring healthcare systems to maximise value by focussing on improving patient outcomes and reducing costs [18]. ABG’s higher-performing IES allows for more streamlined workforce planning, ultimately displacing outdated, administrative-intense rostering strategies. We predict patient outcomes to improve in two main ways. First, the cost savings conferred can be reinvested into bettering patient care. Second, by unlocking the potential of KH’s own workforce not only will safety be enhanced but the efficiency benefits of ‘home-grown’ doctors can be reaped. In the long term, these improved patient outcomes and reduced administrative costs increase value.

By disentangling discrete value-chain activities (see red circle in Fig. 2), we can more accurately analyse the effects of each on value. In this case, by isolating workforce planning as a distinct component of the value chain and employing the IES evaluation grid, we identified areas of improvement in each of the four dimensions, which can help maximise value gains. The value improvements gained by ABG represent an excellent initial step towards demonstrating the importance of evaluating ‘support activities’ to identify sources of inefficiency in healthcare. Additionally, devising assessment frameworks like IES and using research methodologies like ours could be catalytic for evaluating innovations and thus maximising healthcare value. Evidence suggests that this is already happening to some extent. For example, of the 76 innovation studies reviewed by Allen et al., 46% provided assessment of “best practice” innovations, thus highlighting the wider efforts to optimise adoption based on their contributions to healthcare value [48].

In agreement with the DOH’s ‘diffusion pressures’ [14], we identify three source of influence: top-down, horizontal and bottom-up. Top-down pressures were valued less by all informants; an observation encapsulated by ED1’s words that ‘nothing was imposed’. Rather, a ‘concerns-based’ adoption model allowed for considerable consultation with doctors and managers before, during and after the adoption. Whilst meeting the adopters’ concerns is an important prerequisite to successful adoption, continuous feedback is also important; it allows for fluctuations in context affecting user opinion to be caught early and acted upon, thus, retaining user value in the innovation [8, 49] and preventing un-adoption.

A culture receptive to change [50] coupled with inclusive leadership is key for establishing successful value chain linkages. This was evident in KH as the board’s executives regularly used frontline staff’s experiences to inform higher-level decision making. Furthermore, ‘tension for change’ was evident through the rampant frustration amongst managers and doctors with previous locum recruitment strategies [51]. Maximising bottom-up approaches with concurrent policy formulation and implementation is, therefore, critical for healthcare improvements [52].

In keeping with Rogers’s diffusion effect, ‘word of mouth’ has been presented as the most frequent communication channel for diffusion [7]. However, unlike ‘formal dissemination programmes’ [8], this unsystematic method of promotion leaves us sceptical about its potential for diffusion. Despite recent attempts to maximise the spread of innovations throughout the NHS through the ‘NICE Technology Appraisals’, these are strictly limited to medical treatments, thus fall short of appraising equally important, ancillary-related technologies. Although our findings suggest that KH is an example of an ‘opinion leader’ [53], interaction barriers isolating it from future adopters render the diffusion dynamics unfavourable, thus potentially leading to inadequate inter-Trust diffusion. For this reason, we call for KH to join NHS Quest in a new ‘Breakthrough Collaborative’ for sharing the strategy with more Trusts. Considering the increasing importance of ‘joined-up’ care in NHS, this would be beneficial in the current workforce crisis.

Our informants describe simplicity as their most important reason for using the app, demonstrating how explicitly linking an innovation’s function to a specific problem can reduce perceived complexity. We found ‘simplicity’ to be a multi-faceted concept encompassing, among others, features such as higher convenience, better transparency, relative cost-effectiveness and reduced time requirements. All these constituted a highly efficient IES yielding increased perceived advantage. Studies exploring the possibility of substituting traditional communication systems with smartphones and novel software lend support to our findings [54]. We further argue that ABG seems highly compatible with both clinicians and financially constrained Trusts; the former being a group within which smartphone usage has become increasingly popular [55] and both groups finding benefits in smartphone technology solving communication inefficiencies [56, 57]. Therefore, a clear ‘innovation-system’ fit is seen as the values and strategies sought by the NHS align with ABG’s offering [51]. For example, the minimal financial risk associated with the pilot scheme helped KH establish its benefits and identify technical issues that were then addressed accordingly. These, point to the advantages of observability and trialability, both of which we identify as crucial promoters of ABG.

### Limitations

It could be argued that using interviews alone for informing policy or management decisions is inappropriate [58]. To account for this, we deliberately engaged in multi-stakeholder analysis and analyst triangulation to enhance the validity of our findings by allowing cross-checking and a comparison of opinions between different stakeholders. At the time of our study, we were also limited by the fact that only one Trust was piloting the app. However, the app is now being used in other Trusts and we believe a larger mixed methods study examining the impact of the technology in wider settings would be beneficial. A further limitation of this study may be the insufficient exploration of the impact of locum practices on patient safety. The combination of the fact that no patients were recruited in this study and no patient safety data was analysed renders any comments on the topic of patient safety premature. However, incorporating an additional dimension such as patients’ perspectives could have distorted the focus of our objectives and moved our project beyond the intended scope. However, the subject remains pertinent and further research should be directed in this field.

## Conclusion

This study has explored the drivers for locum doctor employment both from the employer’s and the employee’s perspectives with career development, better pay patterns and volatile environment in the NHS being the main ones. These insights may be useful in the future for forming incentives for doctors to turn to permanent employment or internal staff banks and reforming existing training programmes. More importantly, our research has demonstrated the potential of P2PE technologies to support healthcare systems. ABG was found to be a promising solution to KH’s administrative and cost inefficiencies around workforce planning. To this end, the IES model proposed may be useful for more healthcare organisations in evaluating innovations of this kind and better emulating Porter & Teisberg’s VBH model [3]. However, unlike other P2PE-heavy industries, regulatory and legislative barriers of the healthcare sector may hinder its diffusion and subsequent success. Whilst feedback on ABG has generally been positive, the extent of its disruptive influence on the locum industry will require further evaluation in the future.

## Abbreviations

ABG: Alpha beta gamma
App: Application
CQC: Care Quality Commission
Doc Bank: Doctors bank (internal staff bank system)
DOH: Department of Health
F3: Foundation year 3
FOI: Freedom of information
IES: Information exchange system
KH: Kappa Hospital
NHS: National Health Service (UK)
NHSI: NHS improvement
NICE: National Institute for Health and Care Excellence
P2PE: People to people economy
